# Supplementary material for: Reducing bias in coronary heart disease prediction using Smote-ENN and PCA
Source: PLoS One. 2025 Aug 7;20(8):e0327569. doi: 10.1371/journal.pone.0327569 (PMC12331108; doi:10.1371/journal.pone.0327569)
Supplement: Attachment — Submitted filename: Response to reviewers.docx [file pone.0327569.s002.docx]

Response to Reviewers’s Comments

**Manuscript：**PONE-D-25-04242

**Title：**Reducing Bias In Coronary Heart Disease Prediction Using Smote-Enn And Pca

**Author：**Xinyi Wei^1^·Boyu Shi^2^

Authors response to Editor:

Dear Editors

We appreciate to the editor and the reviewers for their so kind feedback on our submission. We have done every possible effort to improve the quality of articles. We have careful modified the paper according to their suggestions. (1) We have revised the introduction section. (2) We supplemented the evaluation indicators of each model training set and proved that no overfitting phenomenon occurred after SMOT-ENN sampling. (3) The test results of people of different genders, ages and educational levels were discussed. (4) Detailed parameters of each model have been supplemented. (5) The added discussion section elaborates in detail on how this data can be applied in practice and the limitations it still has. (6) Regenerate all images into high-resolution and high-definition images and ensure that the terms and graphic references are consistent. (7) The dataset description and data preprocessing sections have been simplified. (8) Use the correct way of literature citation. (9) Detailed revisions were made to the other comments of the reviewers and the entire text was reorganized.

The following are the responses to reviewer #1、Reviewer #2 and Reviewer #3's suggestions.

We hope that our revisions will be satisfactory to render our manuscript suitable for publication in the GRSL.

Once more, thank you very much for taking the time to review our paper and providing detailed and constructive peer review comments.

Best regards!

Sincerely,

Authors.

Reviewer #1、#2 and #3

Thank Reviewer #1、#2 and #3 very much for taking the time to review our manuscript and providing detailed and constructive comments. We tried our best to improve the manuscript and made some changes in the manuscript. These changes will not influence the content and framework of the paper. We appreciate for Editors’ and Reviewers’ warm work earnestly and hope that the corrections will meet with approval. All the revised contents were emphasized in red text in the resubmitted manuscript.

**Responses to reviewer #1's suggestions.**

**Comment 1 to 3-Reviewer #1**

The study lacks a comparison with established CHD risk models such as the Framingham Risk Score or ASCVD risk calculator.

It is unclear how the proposed machine learning models outperform traditional risk prediction models in clinical utility.

The authors should include a direct comparison of model performance against these standard clinical scores.

Authors response to comment 1 to 3:

We appreciate the valuable suggestion that improves the quality of the manuscript. In fact, there is no data on high-density lipoprotein cholesterol (HDL-C) in the dataset provided by kaggle. Therefore, the Framingham Risk Score cannot be calculated and the CHD cannot be calculated through the ASCVD calculator. This dataset is designed to provide a classification dataset for predicting whether a patient has TenyearCHD, for relevant scholars to conduct modeling research.

This is also one of the flaws of this dataset. We mentioned this point in the discussion and provided relevant improvement ideas.

According to the above response, the following content is updated in our revised manuscript.

This study employed the Kaggle public dataset, which has been widely utilized by numerous researchers. However, the dataset has several limitations. First, the dataset contains a high prevalence of missing values, which can significantly affect model performance. Second, there is an imbalance in the distribution of various characteristics, such as smaller samples of certain educational backgrounds or age groups. These issues collectively result in insufficient accuracy of the TenyearCHD prediction model. Additionally, the dataset lacks data on high-density lipoprotein cholesterol (HDL-C), which prevents comparison with the Framingham Risk Score and use of the ASCVD risk calculator. Therefore, future work will focus on using a more comprehensive dataset that includes diverse racial and regional populations. Furthermore, HDL-C data will be collected to develop a high-precision HDL-C prediction model, enabling the full utilization of other parameter information for calculating the Framingham Risk Score.

**Comment 2/3-Reviewer #1**

While SMOTE-ENN is applied to handle data imbalance, it is not clear whether over-sampling led to overfitting, particularly in models like Random Forest and XGBoost.

The study should include a sensitivity analysis on the impact of different resampling techniques (e.g., SMOTE, SMOTE-Tomek) on model performance.

Authors response to comment 2/3:

We apologize for our negligence. Smote-enn is a hybrid sampling method that integrates the advantages of both the SMOTE oversampling and ENN undersampling methods. Judging from the model results, the use of SMOTE can significantly improve the model accuracy, and no overfitting phenomenon has occurred in the model. If SMOTE-ENN is further used, the model accuracy will be higher. To clearly express that this model can avoid overfitting, we provided some indicators of the training set in the unified comparative analysis.

According to the above response, the following content is updated in our revised manuscript.

Through the comparative analysis of the performance of each model under the use of SMODS-ENN and the original imbalanced data (Without), it can be clearly observed that the SMODS-ENN method effectively alleviates the overfitting phenomenon, while the models under the original imbalanced data generally have the risk of overfitting. Under the original data, although the model achieved a relatively high accuracy rate in the training set (such as 90.86% in RF training), its ability to recognize a few classes during testing was extremely poor (Recall was only 7.26%), indicating that the model overly relied on the majority class patterns in the training set and was unable to generalize to real scenarios. By synthesizing samples and cleaning noise, SMOTE-ENN enables the model to learn features more evenly during training. The Recall during testing generally exceeds 90%, and the performance during training and testing improves simultaneously, verifying its effectiveness in suppressing overfitting.

In conclusion, SMODS-ENN optimizes the adaptability of the model to the data distribution through data balance, enhancing the generalization ability while avoiding overfitting. However, the original data causes the model to fall into the local overfitting trap due to category bias.

Table 16. Evaluation results of different models using various data balancing method trees

| Model | Balance Method | Train- Accuracy | Test-Accuracy | Precision | Recall | F1_score | AUC |
| --- | --- | --- | --- | --- | --- | --- | --- |
| DT | Without | 82.35 | 74.76% | 20.78% | 25.81% | 23.02% | 0.54 |
|  | SMOTE-ENN | 90.51 | 85.65% | 86.75% | 90.48% | 88.57% | 0.83 |
| KNN | Without | 02.56 | 83.73% | 35.42% | 13.71% | 19.77% | 0.64 |
|  | SMOTE-ENN | 96.86 | 92.16% | 89.23% | 99.23% | 93.96% | 0.98 |
| SVM | Without | 93.75 | 85.50% | 60% | 2.42% | 4.65% | 0.62 |
|  | SMOTE-ENN | 87.21 | 81.68% | 80.51% | 92.63% | 86.14% | 0.87 |
| XGBoost | Without | 91.76 | 83.02% | 30.77% | 12.90% | 18.18% | 0.64 |
|  | SMOTE-ENN | 95.14 | 91.50% | 92.05% | 94.32% | 93.17% | 0.97 |
| RF | Without | 90.86 | 85.26% | 47.37% | 7.26% | 12.58% | 0.68 |
|  | SMOTE-ENN | 97.34 | 92.16% | 90.57% | 97.39% | 93.85% | 0.98 |

**Comment 4-Reviewer #1**

The study is based on a dataset from Framingham/Kaggle, which does not represent a globally diverse population.

Authors response to comment 4:

Thank you for your attentive comment. In fact, although this dataset is widely used, the range of regions, races, etc. covered by it is insufficient. Therefore, we also mentioned this point in the discussion.

According to the above response, the following content is updated in our revised manuscript.

This study employed the Kaggle public dataset, which has been widely utilized by numerous researchers. However, the dataset has several limitations. First, the dataset contains a high prevalence of missing values, which can significantly affect model performance. Second, there is an imbalance in the distribution of various characteristics, such as smaller samples of certain educational backgrounds or age groups. These issues collectively result in insufficient accuracy of the TenyearCHD prediction model. Additionally, the dataset lacks data on high-density lipoprotein cholesterol (HDL-C), which prevents comparison with the Framingham Risk Score and use of the ASCVD risk calculator. Therefore, future work will focus on using a more comprehensive dataset that includes diverse racial and regional populations. Furthermore, HDL-C data will be collected to develop a high-precision HDL-C prediction model, enabling the full utilization of other parameter information for calculating the Framingham Risk Score.

**Comment 5-Reviewer #1**

Did the authors attempt external validation in an independent cohort? If not, this should be addressed as a major limitation.

Authors response to comment 5:

Thank you for your attentive comment. Thank you for your valuable suggestions. We indeed did not conduct external validation in an independent cohort, which is a limitation of this study. In this study, we mainly used internal cross-validation to evaluate the performance of the model, and did not conduct external validation of the model. Meanwhile, in the conclusion section, the introduction of future related research indicates that our next step will be to conduct research. We plan to collect more independent datasets for external validation to further verify the effectiveness and stability of the diagnostic and predictive framework we proposed. We will also actively explore cooperation with other research institutions to share data resources in order to better carry out external verification work.

Thank you again for your valuable suggestions. We will carefully consider them and make improvements in the subsequent research.

**Comment 6-Reviewer #1**

The authors should discuss potential biases in CHD risk prediction across different ethnicities, ages, and socioeconomic backgrounds.

Authors response to comment 6:

Thank you for your kind comment. We apologize for the lack of clarity in the contribution to this study. In the revised manuscript, we have revised this section. According to our literature, we found that the potential of CNN in rubber plantation AGB estimation was not discovered, and no one had used satellite images with submeter resolution as a data source for AGB estimation. Thank you once again for providing the opinion.

According to the above response, the following content is updated in our revised manuscript.

Furthermore, the test results were segmented and tested according to gender, age and educational level, and the test results are shown in Table 17. First of all, there are certain differences in the performance of the KNN model among different genders. For males, the accuracy rate was 0.903, the precision rate was 0.884, the recall rate was 0.981, and the F1 score was 0.930. The relevant indicators of females performed slightly better, with an accuracy rate of 0.927, a precision rate of 0.889, a recall rate of 0.991, and an F1 score of 0.937. From this, it can be seen that the KNN model performs slightly better on female data and outperforms men in both recall rate and F1 score.

From the perspective of age, the performance of the 30 to 50 age group was the most outstanding, with an accuracy rate of 0.918, a precision rate of 0.877, a recall rate of 1.000, and an F1 score of 0.935. This indicates that the prediction results of this age group are relatively accurate and have a high recall rate. The performance of the 50 to 70 age group was slightly inferior. Although its precision rate was 0.904, the recall rate was 0.977, and the F1 score also remained at a relatively high level (0.939). This might indicate that the model has slight deficiencies in recognizing certain features of the older group, but the overall performance is still acceptable. In terms of educational attainment, the KNN model performs relatively closely at different educational levels. However, for the group with a higher educational attainment (grades 3-4), the F1 score is 0.940, and for the group with a lower educational attainment (grades 1-2), the F1 score is 0.930. This indicates that the group with a higher educational level may be more in line with the recognition pattern of the model. This leads to its even more outstanding performance.

Overall, the KNN model shows certain differences in the segmentation of gender, age and educational level, especially prominent among women and younger groups. In the age group of 30-50 years old, the performance of the RF model was particularly outstanding, with an accuracy rate of 0.950, a precision rate of 0.939, and an F1 score of 0.959, demonstrating the advantages of the RF model in identifying data of this age group. In contrast, the RF model is stable and performs better than the KNN model in most cases, especially. Therefore, the RF model performs more evenly in most subdivision dimensions, especially among groups with higher age and education levels, showing higher accuracy and precision, and is applicable to a wide range of population distributions.

Table 17. Prediction results of KNN and RF under different attribute characteristics

| Model | Attribute | Details | Accuracy | Precision | Recall | F1_score |
| --- | --- | --- | --- | --- | --- | --- |
| KNN | Male | Man | 0.903 | 0.884 | 0.981 | 0.930 |
|  |  | Woman | 0.927 | 0.889 | 0.991 | 0.937 |
|  | Age | 30~50 | 0.918 | 0.877 | 1.000 | 0.935 |
|  |  | 50~70 | 0.917 | 0.904 | 0.977 | 0.939 |
|  | Education | 1-2 | 0.909 | 0.874 | 0.993 | 0.930 |
|  |  | 3-4 | 0.923 | 0.887 | 1.000 | 0.940 |
| RF | Male | Man | 0.913 | 0.905 | 0.970 | 0.936 |
|  |  | Woman | 0.906 | 0.890 | 0.951 | 0.922 |
|  | Age | 30~50 | 0.950 | 0.939 | 0.979 | 0.959 |
|  |  | 50~70 | 0.924 | 0.917 | 0.973 | 0.944 |
|  | Education | 1-2 | 0.918 | 0.900 | 0.974 | 0.936 |
|  |  | 3-4 | 0.923 | 0.886 | 1.000 | 0.940 |

**Comment 7-Reviewer #1**

How were hyperparameters optimized? The methodology mentions grid search, but specific parameters for each model should be detailed.

Authors response to comment 7:

We have made correction according to the Reviewer’s comments. We have explained the parameter results we used in the text for readers to reproduce.

According to the above response, the following content is updated in our revised manuscript .

1. Among them, the min_samples_split in the decision tree is 2 and the min_samples_leaf is 1.
2. Among them, the n_neighbors in KNN is 5, the leaf_size is 30, and p is 2.
3. Among them, the kernel of SVM uses rbf, with a Degree of 3 and C of 1.0.
4. Among them, the n_estimators in XGBoost is 50, max_depth is 3, learning_rate is 0.01, and colsample_bytree is 0.98.
5. Among them, n_estimators in RF is 100, min_samples_split is 2, and min_samples_leaf is 1.

**Comment 8-Reviewer #1**

How was missing data handled? Were imputation techniques used, or were missing values removed?

Authors response to comment 8:

Considering the Reviewer’s suggestion. In the revised manuscript, details for specifically filling in the missing values have been provided.

According to the above response, the following content is updated in our revised manuscript .

Missing values of variables complicate the data analysis process and may skew the final results . This thesis uses the common mode filling method to process missing values based on label data such as education and male. For cigsPerDay, BPMeds, totChol, BMI, heartRate, and glucose, these feature variables are numerical values that are filled in using the mean-filling approach. The goal of the processing described in this research is to keep as much data as feasible while preserving the statistical properties of the data. Specifically, for classification features (such as education and male), the mode is used to fill in the missing values. For numerical features (such as cigsPerDay, BPMeds, etc.), use the mean to fill in the missing values.

**Comment 9-Reviewer #1**

What statistical tests were used to compare model performances? The authors should clarify if paired t-tests or DeLong’s test for AUC comparison were applied.

Authors response to comment 9:

Thank you for your attentive comment. To facilitate readers' reproduction, for all models, we set the random seed to 42. Therefore, the results of each run are the same and there are no differences. If necessary, we can, in the next revision, refrain from setting a fixed random seed and conduct multiple experiments to explore the stability.

**Comment 10-Reviewer #1**

The manuscript does not discuss how the model would be integrated into clinical practice.

Authors response to comment 10:

We appreciate the valuable suggestion that improves the quality of the manuscript. In the revised manuscript, the first paragraph of our discussion briefly describes the contribution of this study and how it can be applied in combination with clinical practice.

According to the above response, the following content is updated in our revised manuscript.

The TenyearCHD prediction model developed in this study holds significant potential for application in clinical practice. It can serve as a valuable tool for healthcare professionals in assessing the likelihood of patients developing cardiovascular disease within a ten-year period. By providing early risk stratification, the model enables physicians to identify high-risk individuals promptly, facilitating timely interventions and personalized treatment plans. This can lead to improved patient outcomes, more efficient allocation of healthcare resources, and cost-effective management of cardiovascular disease. Furthermore, the model's ability to predict TenyearCHD can aid in patient education and counseling, empowering individuals to make informed decisions about lifestyle modifications and adherence to preventive measures. Overall, the integration of this machine learning prediction model into clinical workflows can enhance the prevention and management of cardiovascular disease, contributing to better public health.

**Comment 11-Reviewer #1**

The authors should provide decision thresholds for CHD risk classification (e.g., at what probability cutoff should an individual be flagged as high risk?).

Authors response to comment 11:

Considering the Reviewer’s suggestion. In the revised manuscript. We added explanations in the revised manuscript. According to the background of clinical practice, a threshold of 0.5 is very appropriate. 0.5 represents a 50% risk and is the standard of "high risk".

**Comment 12-Reviewer #1**

The authors focus solely on data-driven methods but should discuss other potential approaches such as genetic risk scores, deep learning models, and wearable device-based risk prediction.

Authors response to comment 13:

We appreciate the valuable suggestion that improves the quality of the manuscript. In the revised manuscript, the last paragraph of our discussion mentioned that the deployment of the diagnostic model to micro-devices would be considered. Meanwhile, the deep learning model capable of end-to-end diagnosis is also the model that will be used in the next step of research.

According to the above response, the following content is updated in our revised manuscript.

Finally, deep learning technology has demonstrated excellent performance when dealing with complex and large-scale data. Therefore, in CHD risk prediction, through automated feature extraction and nonlinear relationship modeling, it can improve the accuracy and sensitivity of prediction to a certain extent. In addition, data collection based on wearable devices makes real-time monitoring possible. By integrating sensor technology and deep learning models, it is possible to assess an individual's health status more accurately and predict potential disease risks in a timely manner. Consider embedding these advanced models into micro-medical devices, which will provide portable CHD detection tools for relevant personnel. By integrating deep learning models with wearable devices, users can monitor their cardiovascular health at any time in their daily lives, achieving the goals of early warning and personalized intervention, thereby effectively reducing the risk of heart attacks and improving public health.

**Responses to** **reviewer #2's suggestions.**

**Comment 1-Reviewer #2**

Some explanations, such as dataset description and missing data handling, could be more concise and precise.

Authors response to comment 1:

We appreciate the valuable suggestion that improves the quality of the manuscript. In the revised manuscript, dataset description and missing data processing section be more concise and precise. Specifically, we strictly adjusted the writing format of "text first, then table". Then classify and organize the feature tables, and also delete the variables that are not missing in Table 4 to make these two parts clearer.

According to the above response, the following content is updated in our revised manuscript.

The dataset used in this dissertation’s investigation of coronary heart disease is sourced from the "framingham.csv" database on Kaggle. It originates from an ongoing cardiovascular study involving residents of Framingham, Massachusetts. The primary goal of data collection is to

predict whether individuals are likely to develop coronary heart disease within the next 10 years (Kaggle, n.d.). The dataset comprises 4,238 samples and 16 variables, including one target variable and 15 feature variables. The target variable is used to forecast whether participants will experience coronary heart disease within a ten-year period. As shown in Table 3, In this experiment, non-numeric data needs to be converted to numeric data because numeric data. The dataset used in this project contains 15 feature variables and 1 target variable, of which 6 are binary variables, 1 is a multi-category variable, and 8 are numeric variables.

According to the above response, the following content is updated in our revised manuscript.

Data analysis reveals that the following feature variables contain missing values: Education, Cigarettes per Day (cigsPerDay), Use of Blood Pressure Medication (BPMeds), Total Cholesterol (totChol), Body Mass Index (BMI), Heart Rate (heartRate), and Glucose Level (glucose). Table 4 presents the number and percentage of missing values for each feature. The missing value of the glucose level characteristic is close to 10%, which will have a significant impact on the diagnostic performance of the model.

Table 4. Missing values and percentages for each attribute

| Attribute | Number of missing values | Percentage of missing values（%） |
| --- | --- | --- |
| education | 105 | 2.48 |
| cigsPerDay | 29 | 0.68 |
| BPMeds | 53 | 1.25 |
| totChol | 50 | 1.18 |
| BMI | 19 | 0.45 |
| heartRate | 1 | 0.02 |
| glucose | 388 | 9.15 |

**Comment 2-Reviewer #2**

Clarifying the rationale behind certain choices, like encoding methods and feature selection, would enhance readability.

Authors response to comment 2:

Thank you for your attentive comment. Feature selection is a very important task. We haven't elaborated on its working principle in detail before. In the revised manuscript, We have refined the description part of the feature selection used. Meanwhile, we have integrated the redundant images and repetitive table descriptions in order to better present the results of feature selection.

According to the above response, the following content is updated in our revised manuscript.

The Chi-square test was used to evaluate the connection between the target variable (TenYearCHD) and each feature. The chi-square test is carried out in the following manner: Create a contingency table using the observed values of the characteristics and target variables. The predicted frequency for each cell is then computed, which is the frequency of observations in that cell, assuming that the feature and target variable are independent. Finally, the chi-square statistic is calculated using the formula:

| $X^{2}$=$\sum\frac{{（O-E）}^{2}}{E}$ | (3) |
| --- | --- |

where $X^{2}$ is the chi-square statistic, O is the observed frequency, and E is the expected frequency.

The chi-square statistic for the whole contingency table is obtained by adding all cells together. A higher chi-square statistic score suggests that the characteristic predicts the target variable better. Figure 1 (a) illustrates each feature's relevance score from the table below. On the coronary heart disease data set, the correlation between each feature and the risk of coronary heart disease was evaluated, and the chi-square test was used to rank the value of the characteristics. To achieve the purpose of quickly screening features. Perform a chi-square test on the data set's 15 category feature variables using Python. Table 5 and Figure 1(b) displays the results of the chi-square test on the characteristic variable and the following conclusions.

Table 5. Characteristic variable scores for the chi-square test

| Feature | Conclusion | Score |
| --- | --- | --- |
| sysBP | The crucial conclusion is that systolic blood pressure has a significant role in predicting the probability of coronary heart disease. Increased systolic blood pressure is usually associated with cardiovascular problems. | 728.292522 |
| glucose | Diabetes, a known risk factor for coronary heart disease, isindicated by high glucose levels. | 390.825416 |
| age | As the risk of cardiovascular disease grows with age, this is an important element in risk prediction. | 318.597444 |
| totChol | It is a key predictor of coronary heart disease due to its close link with cardiovascular health. | 234.305978 |
| cigsPerDay | Consider the potential negative health impacts of smoking, which has been linked to an increased risk of heart disease. | 221.680719 |
| diaBP | Although somewhat less relevant than systolic blood pressure, diastolic blood pressure is nevertheless an essential blood pressure measure in prediction models. | 153.090954 |
| prevalentHyp | Hypertension is a prominent risk factor for cardiovascular disease, and the feature scores show that it has a role in the model's prediction. | 92.167857 |
| diabetes | Diabetes is related to a variety of cardiovascular problems and is a key factor in predicting coronary heart disease risk, as evidenced by trait scores. | 39.103710 |
| BPMeds | It might be a hypertensive response, or it could be an indication of cardiovascular disease.  It is a risk factor for coronary artery disease, and men are more vulnerable than women. | 30.578341 |
| male | A history of stroke implies that a person is more likely to develop cardiovascular problems. | 18.915212 |
| prevalentStroke | The model's importance score is low, yet it is routinely used to analyse the influence of weight on health, particularly the risk of cardiovascular disease. | 16.095638 |
| BMI | Although educational level is related to health behaviours and outcomes, it does not appear to be a substantial predictor in our model. | 15.177679 |
| education | Although heart rate is an important measure of cardiovascular health, it plays little role in this model. | 6.271609 |
| heartRate | The model gave less weight to whether or not a person smoked, suggesting that daily cigarette intake is more powerful than simple smoking habits in predicting the risk of coronary heart disease. | 4.2334763 |
| currentSmoker | Diabetes, a known risk factor for coronary heart disease, isindicated by high glucose levels. | 0.811603 |

**Comment 3-Reviewer #2**

The discussion on standardization, normalization, and PCA could be expanded slightly for better clarity.

Authors response to comment 3:

Considering the Reviewer’s suggestion. We did not take this part into account when we wrote. In the revised manuscript, we checked whether these three parts were described clearly and found that the description of PCA was relatively brief and there were no visualization results. Therefore, in the revised manuscript, we examined the parts of data standardization and normalization. Meanwhile, the PCA part is supplemented with emphasis.

According to the above response, the following content is updated in our revised manuscript.

This research applies PCA to reduce the dimensionality of the coronary heart disease dataset. PCA transforms original data into linearly uncorrelated principal components, capturing most data variance with fewer variables. Its benefits include reducing feature numbers, simplifying data structure, improving analysis efficiency, lowering computational costs, speeding up model training and prediction, minimizing data noise, and enhancing prediction accuracy. As shown in Figure 2, we conducted PCA visualization. The variance interpretation rates of each principal component can be understood through the visualization results. In Figure 2, the left figure shows the visualization results of PC1-PC2, and the right figure shows the visualization results of PC3-PC4. It can be found that the sum of the variance explanations from PC1 to PC4 is 88%(> 85%). However, the specific dimension to which it should be reduced is related to the model. Therefore, the specific dimension situation will be explained in the subsequent experiments.

Figure 2. Visualization of PCA results

**Comment 4-Reviewer #2**

Minor inconsistencies in terminology and figure references should be addressed for a smoother flow.

Authors response to comment 4:

We have made correction according to the Reviewer’s comments. In the revised manuscript, We have checked the image and the related reference positions, and also examined whether the image is clear and other issues. Thank you again to the reviewers for their suggestions.

**Comment 5-Reviewer #2**

Explaining what would be the effect and impact i.e. how it can be employed in clinical practice will add into the value of your manuscript

Authors response to comment 5:

We appreciate the valuable suggestion that improves the quality of the manuscript. In the revised manuscript, the first paragraph of our discussion briefly describes the contribution of this study and how it can be applied in combination with clinical practice.

According to the above response, the following content is updated in our revised manuscript.

The TenyearCHD prediction model developed in this study holds significant potential for application in clinical practice. It can serve as a valuable tool for healthcare professionals in assessing the likelihood of patients developing cardiovascular disease within a ten-year period. By providing early risk stratification, the model enables physicians to identify high-risk individuals promptly, facilitating timely interventions and personalized treatment plans. This can lead to improved patient outcomes, more efficient allocation of healthcare resources, and cost-effective management of cardiovascular disease. Furthermore, the model's ability to predict TenyearCHD can aid in patient education and counseling, empowering individuals to make informed decisions about lifestyle modifications and adherence to preventive measures. Overall, the integration of this machine learning prediction model into clinical workflows can enhance the prevention and management of cardiovascular disease, contributing to better public health.

**Responses to reviewer #3's suggestions.**

**Comment 1-Reviewer #3**

Kindly indicate all your in-text references or citations as superscripts or preferably in square bracket [...] as recommended by PLOS ONE to differentiate it from the main text.

Authors response to comment 1:

Thank you for your attentive comment. This is our mistake. There are a large number of problems with the citation format of the literature in Table 1 and Table 2. In the revised manuscript, we checked all the literature citations. And we have marked the cited literature with the […] symbol and emphasized it with blue text.

For example.

Before modification:

1. In clinical decision support systems, it enhances model accuracy 18.
2. Wu (2022) 21

After modification:

1. In clinical decision support systems, it enhances model accuracy **[18]**.
2. Wu (2022) **[21]**

**Comment 33-Reviewer #3**

The in-text reference in Line 45, 46 and 53 are quite misleading. It is best if you separate them with commas (,).

Authors response to comment 2:

Considering the Reviewer’s suggestion. We have adjusted this part and separated different documents with commas (,).

**Comment 3-Reviewer #3**

Check the word "practiceto" in Line 51.

Authors response to comment 3:

We apologize for our negligence. In the revised manuscript, we have adjusted “Practicesto” to “practices to”

**Comment 4-Reviewer #3**

Line 53 - There is an error information "Error! Reference source not found." Kindly fix that.

Authors response to comment 4:

Thank you for reading carefully and putting forward the suggestions. We readjusted the reference in line 53 to ensure its correctness.

**Comment 5-Reviewer #3**

The citation "(Kaggle, n.d.)" introduces another reference which violates PLOS ONE's citation 6 / 8 recommendation. Moreover, there is no date.

Authors response to comment 5:

Thank you for your attentive comment. We found that our previous manuscript did not correctly refer to the entire dataset. In the revised manuscript, we added the corresponding DOI as required by Kaggle.

According to the above response, the following content is updated in our revised manuscript.

The primary goal of data collection is to predict whether individuals are likely to develop coronary heart disease within the next 10 years (<https://doi.org/10.34740/kaggle/dsv/3493583>).

**Comment 6-Reviewer #3**

Line 146 - the statement is not clear

Authors response to comment 6:

Thank you and the other reviewers for the problems that occurred in this statement and this part of the description, as well as the suggestions for revision provided. In the revised manuscript, we reorganized this section.

According to the above response, the following content is updated in our revised manuscript.

The chi-square statistic for the whole contingency table is obtained by adding all cells together. A higher chi-square statistic score suggests that the characteristic predicts the target variable better. Figure 1 (a) illustrates each feature's relevance score from the table below. On the coronary heart disease data set, the correlation between each feature and the risk of coronary heart disease was evaluated, and the chi-square test was used to rank the value of the characteristics to achieve the purpose of quickly screening features. Perform a chi-square test on the data set's 15 category feature variables using Python. Table 5 and Figure 1(b) displays the results of the chi-square test on the characteristic variable and the following conclusions.

**Comment 7-Reviewer #3**

Table 6 is difficult to follow. Kindly format it well

Authors response to comment 7:

We appreciate the valuable suggestion that improves the quality of the manuscript. In the previous Table 6, we used incorrect line breaks and redundant expressions. In the revised and reorganized manuscript, Table 5 and Table 6 were combined, thereby expressing the purpose of this subsection more clearly.

According to the above response, the following content is updated in our revised manuscript.

Table 5. Characteristic variable scores for the chi-square test

| Feature | Conclusion | Score |
| --- | --- | --- |
| sysBP | The crucial conclusion is that systolic blood pressure has a significant role in predicting the probability of coronary heart disease. Increased systolic blood pressure is usually associated with cardiovascular problems. | 728.292522 |
| glucose | Diabetes, a known risk factor for coronary heart disease, is indicated by high glucose levels. | 390.825416 |
| age | As the risk of cardiovascular disease grows with age, this is an important element in risk prediction. | 318.597444 |
| totChol | It is a key predictor of coronary heart disease due to its close link with cardiovascular health. | 234.305978 |
| cigsPerDay | Consider the potential negative health impacts of smoking, which has been linked to an increased risk of heart disease. | 221.680719 |
| diaBP | Although somewhat less relevant than systolic blood pressure, diastolic blood pressure is nevertheless an essential blood pressure measure in prediction models. | 153.090954 |
| prevalentHyp | Hypertension is a prominent risk factor for cardiovascular disease, and the feature scores show that it has a role in the model's prediction. | 92.167857 |
| diabetes | Diabetes is related to a variety of cardiovascular problems and is a key factor in predicting coronary heart disease risk, as evidenced by trait scores. | 39.103710 |
| BPMeds | 1.It might be a hypertensive response, or it could be an indication of cardiovascular disease.  2. It is a risk factor for coronary artery disease, and men are more vulnerable than women. | 30.578341 |

**Comment 8-Reviewer #3**

All the figures in the work are blurry and difficult to read from. Kindly provide a sharper image which can easily be referred to

Authors response to comment 8:

We apologize for our negligence. We have modified all the images to ensure that their DPI is above 300, guaranteeing compliance with the journal's inclusion requirements.

**Comment 9-Reviewer #3**

The work needs major reorganization in order to clearly identify the methods used to assess the various machine learning models for predicting coronary heart disease, the results and the discussion with adequate synthesis of existing literature

Authors response to comment 9:

Thank you for your attentive comment. Our manuscript has many problems and significant adjustments were made during the revision process, including but not limited to the adjustment of chapter order, as well as the addition and reduction of chapters. It ensures that the article clearly expresses the data situation, data preprocessing methods, feature extraction methods, sampling techniques and modeling diagnosis results used in this study.

We sincerely thank the reviewers for the time and effort they have devoted to evaluating our manuscripts. We look forward to any additional feedback or suggestions to further refine our research.

Yours sincerely,

Xinyi Wei

May 6, 2025

Universiti Malaya, Institute For Advanced Studies.

Email: wxye1848306171@outlook.com
